# Antioxidant and cytotoxic activities of three species of tropical seaweeds

**DOI:** 10.1186/s12906-015-0867-1

**Published:** 2015-09-29

**Authors:** Yin Yin Chia, M S Kanthimathi, Kong Soo Khoo, Jayakumar Rajarajeswaran, Hwee Ming Cheng, Wai Sum Yap

**Affiliations:** Department of Molecular Medicine, Faculty of Medicine, University of Malaya, 50603 Kuala Lumpur, Malaysia; University of Malaya Centre for Proteomics Research (UMCPR), University of Malaya, Kuala Lumpur, Malaysia; Department of Chemical Science, Faculty of Science, Universiti Tunku Abdul Rahman (Kampar campus), Jalan Universiti, Bandar Barat, 31900 Kampar, Perak Malaysia; Department of Physiology, Faculty of Medicine, University of Malaya, 50603 Kuala Lumpur, Malaysia; Department of Biotechnology, Faculty of Applied Sciences, UCSI University, 56000 UCSI Heights, Kuala Lumpur, Malaysia

**Keywords:** *Padina tetrastromatica*, *Caulerpa racemosa*, *Turbinaria ornata*, Cytotoxic activity, MTT assay, Antioxidant activity, Antioxidant enzyme activity, LC-MS, Caspase activity, DNA fragmentation

## Abstract

**Background:**

Three species of seaweeds (*Padina tetrastromatica*, *Caulerpa racemosa* and *Turbinaria ornata*) are widely consumed by Asians as nutraceutical food due to their antioxidant properties. Studies have shown that these seaweeds exhibit bioactivities which include antimicrobial, antiviral, anti-hypertensive and anticoagulant activities. However, investigations into the mechanisms of action pertaining to the cytotoxic activity of the seaweeds are limited. The aim of this study was to determine the antioxidant and cytotoxic activities of whole extracts of *P. tetrastromatica*, *C. racemosa* and *T. ornata,* including the cellular events leading to the apoptotic cell death of the extract treated-MCF-7 cells. Bioassay guided fractionation was carried out and the compounds identified.

**Methods:**

Powdered samples were sequentially extracted for 24 h. Their antioxidant activities were assessed by the DPPH radical, superoxide, nitric oxide and hydroxyl radical scavenging assays. The cytotoxic activity of the extract-treated MCF-7cells was assessed using the MTT assay. The most potent fraction was subjected to bioassay guided fractionation with column chromatography. All the fractions were tested for cytotoxic activity, caspase activity and effect on DNA fragmentation.

**Results:**

All three seaweeds showed potent radical scavenging activities in the various assays. The activity of the cellular antioxidant enzymes, superoxide dismutase, catalase and glutathione reductase, in MCF-7 cells, decreased in a time-dependent manner. The partially purified fractions exhibited higher cytotoxic activity, as assessed by the MTT assay, than the whole extracts in the breast adenocarcinoma cell line, MCF-7. LC-MS analysis revealed the presence of bioactive alkaloids such as camptothecin, lycodine and pesudopelletierine.

**Conclusion:**

Based on the results obtained, all three seaweeds are rich sources of enzymatic and non-enzymatic antioxidants which could contribute to their reported medicinal benefits.

## Background

Cancer is a health issue causing utmost concern and continuing to be one of the leading causes of mortality worldwide, accounting for 7.9 million deaths in 2007. This figure is predicted to continue to increase, with an estimated 12 million deaths in 2030. According to the World Cancer Report, the worldwide burden of cancer is expected to rise to 22 million new cases annually within the next two decades while global cancer deaths are predicted to rise from an estimated 8.2 million annually to 13 million per year [1]. Breast cancer tops the list as the most prevalent cancer today, followed by prostate, colon or rectal, lung and ovarian cancers [2].

Despite rapid advancements in medicine, a universally effective treatment for cancer has yet to be found. Conventional cancer treatments include surgery, radiation and chemotherapy, but these modes of treatment do not always prevent metastasis and fatality. Therefore, there exists a need to search for alternative treatments for cancer.

Marine organisms inhabit intertidal areas which are considered harsh environments due to tidal fluctuations [3, 4]. These environmental stresses would ultimately lead to the generation of free radicals and reactive oxygen species (ROS). However, marine organisms are usually protected and unharmed despite the presence of these stresses due to the presence of protective mechanisms mediated by enzymes or antioxidant compounds [3, 5].

The diversity of marine organisms has become an inspiration for researchers to identify novel marine natural products that could eventually be developed into therapeutics or pharmaceutical products. In fact, many structurally diverse natural products isolated from marine organisms are reported to exhibit an astounding array of bioactivities, particularly anticancer activity against multiple tumour types, antibiotic, antiviral, antioxidant and anti-inflammatory activities [6, 7]. More than 3000 new substances have been identified from marine organisms over the past three decades, showing the great potential of the ocean as a source of novel chemical compounds [8, 9].

Cells contain natural antioxidants as a protective mechanism to combat the deleterious effects of ROS [10]. Examples ofnatural antioxidants in the human body are the enzymes, catalase and hydroperoxidase, which catalyze the conversion of hydrogen peroxides and hydroperoxides to non-radical forms [11]. Natural antioxidants are potential pharmaceutical and therapeutic agents [12]. The pharmacological and physiological actions of phenolic compounds are primarily ascribed to their antioxidant capacity, free radical scavenging ability and modulation of gene expression [13].

This study aimed to evaluate the cytotoxic and antioxidant activities of three seaweeds, *Padina tetrastromatica*, *Caulerpa racemosa* and *Turbinaria ornata. P. tetrastromatica* is a brown alga which grows in tropical waters. It is usually sprinkled as dried flakes on food as seasoning and also serves as a table salt replacement for hypertensive patients. Studies have shown that *P. tetrastromatica* has high anticoagulant and antiviral activities due to the presence of alginic acid [14]. *C. racemosa* is a green alga normally found in tropical and subtropical regions. It is regularly eaten cooked or consumed raw as a salad in Thailand. Besides its use as animal feed, it is also used in humans for the treatment of hypertension and rheumatism [15]. *T. ornata* is a brown alga commonly found in disturbed coral reef crests. These three seaweeds are used as folk medicaments for various ailments. They have been reported to exhibit a broad spectrum of bioactivities, including antimicrobial activity, treatment for parasitic infections, anticoagulant activity, antiviral activity and antitumor activity [16].

Seaweeds are rich sources of vitamins A, B1, B12, C, D and E, riboflavin, folic acid, pantothenic acid and minerals such as sodium, potassium, calcium and phosphorus. They contain most of the essential amino acids required for the maintenance of health. In addition, seaweeds contain more than 54 trace elements which are vital for physiological functions in humans, in quantities significantly exceeding those in vegetables and other terrestrial plants [16]. Seaweeds are also used medicinally in China for the treatment of liver diseases, swelling, cysts, phlegm and enlarged thyroid glands. During the 18^th^ century, kelp was discovered as a rich source of iodine in the diet and is used to treat enlarged thyroid glands [15]. Since the early 1980s, several types of algae have been marketed as supplements. Spirulina, Chlorella and certain blue-green algae are among the examples of supplements sold worldwide [16].

Despite their wide usage in traditional medicine, relatively little is known about the bioactivity of these seaweeds and to date, scientific documentation of their bioactivities is scanty to virtually none. We determined the cytotoxic effect of *P. tetrastromatica*, *C. racemosa* and *T. ornata* on the breast cancer cell line, MCF-7, by the MTT (3-(4,5-dimethylthiazol-2-yl)-2,5-diphenyltetrazolium bromide) assay. We also determined their antioxidant activities by the 1,1-diphenyl-2-picrylhydrazyl (DPPH) radical, superoxide anion radical, nitric oxide and hydroxyl radical scavenging assays. The total phenolic content (TPC) and total flavonoid content (TFC) were also determined. The antioxidant enzymes, superoxide dismutase (SOD), catalase (CAT) and glutathione reductase (GR) were assayed in MCF-7 cells treated with each of the three algae. Caspase activity assays and DNA fragmentation analysis were also carried out to determine the mechanism of cell death.

## Methods

### Extract preparation

The three seaweeds, *P. tetrastromatica*, *C. racemosa* and *T. ornata*, were collected from the coastal regions of Port Dickson, Malaysia. They were identified by Khoo Kong Soo and voucher specimens for the three seaweed samples were deposited at the Department of Chemical Science, Faculty of Science, Universiti Tunku Abdul Rahman, Kampar, Malaysia, under numbers UTAR/FSc/10/001; UTAR/FSc/10/002 and UTAR/FSc/10/003, respectively. They were washed, cleaned and cut into smaller pieces before being freeze-dried and subsequently ground into powder. The powders were extracted sequentially using solvents of different polarities, namely, hexane, dichloromethane (DCM), ethyl acetate (EA), acetone and methanol. Ground materials (25 g) were extracted with 500 mL of the solvent for 24 h at room temperature. The same extraction procedure was repeated thrice before changing to a solvent of higher polarity. The filtrates were filtered through Whatman No. 1 filter paper and subsequently concentrated with a rotary evaporator. The concentrated whole extracts were dissolved in 10 % dimethyl sulfoxide (DMSO). The percentage of yield of each extract was calculated using the following eq. (1).

1 $$ \mathrm{Percentage}\kern0.5em \mathrm{of}\kern0.5em \mathrm{yield}=\frac{\mathrm{Final}\kern0.5em \mathrm{weight}\kern0.5em \mathrm{of}\kern0.5em \mathrm{dried}\kern0.5em \mathrm{extract}}{\mathrm{Initial}\kern0.5em \mathrm{weight}\kern0.5em \mathrm{of}\kern0.5em \mathrm{powder}}\times 100\% $$

### Cell culture

The normal human breast cell line, 184B5, and the human breast adenocarcinoma cell line, MCF-7, were purchased from American Type Culture Collection (ATCC, VA). The cells were grown in RPMI medium, supplemented with 10 % fetal bovine serum (FBS), 1 % (v/v) penicillin-streptomycin antibiotics and 1 % (v/v) glutamine (all from Sigma-Aldrich). The cells were maintained at 37 °C in a humidified incubator containing 5 % CO_2_

### Cytotoxic activity

The extracts were dissolved in DMSO and added to medium to make the final concentration of DMSO less than 1 % per well. Cell viability was determined by the MTT assay. 184B5 and MCF-7 cells were plated at a density of 1 × 10^4^ cells/well in a 96-well plate. The cells were cultured for 24 h and subsequently treated with the extracts at doses of 100–500 μg/mL for 48 h. Doxorubicin was used as the positive control while vehicle control DMSO was used as the negative control. After incubation, 10 μL/well of MTT was added and further incubated for 4 h at 37 °C. Spent medium was aspirated and the insoluble formazan dye was dissolved with DMSO. The absorbance of the colored product was read at 595 nm.

### Determination of total phenolic content (TPC)

The TPC was determined by a method described previously [17] with slight modifications in the concentration of Na_2_CO_3_. The assay involved Folin-Ciocalteu (FC) reagent, gallic acid as the standard and quercetin and rutin as the positive controls. One mL of 10 % FC reagent was added to 20 μL of extract or standard. The reagents were mixed well and incubated for 5 min before adding 700 μL of 10 % Na_2_CO_3._ The solutions were further incubated for 2 h before reading the absorbance at 765 nm. Gallic acid in the range of 20–200 mg/L was used to construct a calibration curve. TPC values were expressed as mg gallic acid equivalent (GAE)/g dried weight. The experiments were performed in triplicate.

### Determination of total flavonoid content (TFC)

The aluminum chloride colorimetric method [18] was used in the determination of TFC. Quercetin was used as the standard while catechin was used as the positive control. Sixty μL of methanol was mixed with 20 μL of extract or standard, 4 μL of 10 % aluminum chloride, 4 μL of 1.0 M potassium acetate and 122 μL of MilliQ water. The mixtures were incubated at room temperature for 30 min before reading the absorbance at 415 nm. A calibration curve was prepared using catechin at concentrations of 12.5 - 100 μg/mL in methanol. TFC was expressed as mg of catechin equivalents/g dried weight. All experiments were performed in triplicate.

### Determination of antioxidant activities

#### 1,1-diphenyl-2-picrylhydrazyl (DPPH) Radical Scavenging Activity

1,1-diphenyl-2-picrylhydrazyl radical was used in the evaluation of radical scavenging activity of the extracts as described [19] with minor changes in the DPPH concentration. The reaction mixtures, containing 120 μL of 0.04 mg/mL DPPH solution in methanol, were mixed with 20 μL of different concentrations (25–400 μg/mL) of the extracts and shaken vigorously before being incubated in the dark for 20 min. Quercetin and rutin were the positive controls. Reduction in the absorbance of DPPH was measured against a blank at 517 nm. The radical scavenging activity was calculated using eq. (2).

2 $$ \mathrm{Percentage}\ \mathrm{of}\ \mathrm{DPPH}\ \mathrm{radical}\ \mathrm{scavenging}\ \left(\%\right)=\left({\mathrm{A}}_{\mathrm{blank}}\hbox{-} {\mathrm{A}}_{\mathrm{samples}/\mathrm{positive}\kern0.5em \mathrm{controls}}\right)/{\mathrm{A}}_{\mathrm{blank}}\times 100 $$

Where A_blank_ and A_sample/positive control_ denote the absorbance of blank and absorbances of samples or positive controls, respectively.

#### Superoxide anion radical scavenging activity

The superoxide anion scavenging activity was assayed according to a method described earlier [20] with slight modifications in the nitroblue tetrazolium (NBT) and phenazine methosulphate (PMS) concentrations. The reaction mixture containing 25 μL of NBT solution (150 μM NBT in 100 mM phosphate buffer, pH 7.4), 2 μL of PMS solution (60 μM PMS in 100 mM phosphate buffer, pH 7.4) and 20 μL of NADH solution (468 μM in 100 mM phosphate buffer, pH 7.4) was added to different concentrations (25–400 μg/mL) of the extracts. The mixture was incubated in the dark for 10 min at 25 °C and the absorbance read at 560 nm. Quercetin and rutin were the positive controls. All experiments were done in triplicate and results were expressed as percentage inhibition of superoxide anion radical using eq. (3).

3 $$ \mathrm{Percentage}\ \mathrm{of}\ \mathrm{superoxide}\ \mathrm{anion}\ \mathrm{radical}\ \mathrm{scavenging}\ \left(\%\right)=\left({\mathrm{A}}_{\mathrm{blank}}\hbox{--}\ {\mathrm{A}}_{\mathrm{samples}/\mathrm{positive}\ \mathrm{control}}\right)/{\mathrm{A}}_{\mathrm{blank}} \times 100\% $$

#### Nitric oxide scavenging activity

The nitric oxide scavenging activity was determined using a previously described method [21]. 10 μL of 10 mM sodium nitroprusside (SNP) in phosphate buffer was mixed with 10 μL of different concentrations (25–400 μg/mL) of extracts. The mixture was incubated in the dark at room temperature for 2.5 h. Quercetin and rutin were used as positive controls. After the incubation period, 40 μL of sulphanilic acid reagent (0.33 % sulphanilic acid in 20 % glacial acetic acid) was added to the mixture and further incubated for 5 min, after which 40 μL of 0.1 % naphthyl ethylene diamine dihydrochloride was added, mixed and incubated for another 30 min at 25 °C. The absorbance of the chromophore formed was read at 540 nm. All determinations were performed in triplicate and results were expressed as percentage of nitric oxide scavenged by using eq. (4).

4 $$ \mathrm{Percentage}\ \mathrm{of}\ \mathrm{nitric}\ \mathrm{oxide}\ \mathrm{scavenged}\ \left(\%\right)=\left({\mathrm{A}}_{\mathrm{blank}}\hbox{--}\ {\mathrm{A}}_{\mathrm{samples}/\mathrm{positive}\ \mathrm{control}}\right)/{\mathrm{A}}_{\mathrm{blank}} \times 100\% $$

#### Hydroxyl radical scavenging activity

The site specific hydroxyl radical scavenging assay was determined as described [22]. The reaction mixture containing 23.8 μL of 100 mM FeCl_3_ solution, 23.8 μL of 1.25 mM H_2_O_2_ solution, 23.8 μL of 2.25 mM deoxyribose and 23.8 μL of 100 mM ascorbic acid was added to 5 μL of different concentrations (25–400 μg/mL) of extracts. The mixture was incubated at 37 °C for 1 h after which 100 μL of 0.5 % of thiobarbituric acid (TBA) in 25 mM NaOH and 100 μL of 2.8 % trichloroacetic acid (TCA) were added. The resulting mixture was then boiled at 100 °C for 15 min and subsequently cooled on ice before taking the absorbance readings at 550 nm. Quercetin and rutin were used as positive controls. All determinations were performed in triplicate and results were expressed as percentage of hydroxyl radical scavenging activity as calculated by eq. (5).

5 $$ \mathrm{Percentage}\ \mathrm{of}\ \mathrm{hydroxyl}\ \mathrm{radical}\ \mathrm{scavenged}\ \left(\%\right)=\left({\mathrm{A}}_{\mathrm{blank}}\hbox{--}\ {\mathrm{A}}_{\mathrm{samples}/\mathrm{positive}\ \mathrm{control}}\right)/{\mathrm{A}}_{\mathrm{blank}} \times 100\% $$

### Preparation of cell extracts for antioxidant enzyme assays

The methanolic extracts of all three samples were selected for antioxidant enzyme assays based on the results of TPC, TFC and DPPH radical scavenging activity. The cell extracts were prepared according to described methods [23] with slight modifications in the lysis buffer (Tris/HCl 20 mM and 0.2 % Triton X-100) and extract concentrations used. Cells were seeded in 25 cm^2^ flasks and incubated for 24 h. Extracts (400 μg/mL) were added and incubated for another 8 h, 16 h and 24 h. After incubation, the cells were washed with phosphate buffered saline (PBS) and harvested with a cell scraper. Cells were centrifuged at 1500 rpm for 10 min, pellets resuspended in 1 mL lysis buffer and sonicated thrice on ice. Finally, the cells were centrifuged for 15 min at 3000 rpm at 4 °C and the supernatant stored at −80 °C.

### Determination of antioxidant enzyme activities

#### Superoxide dismutase (SOD) assay

The SOD activity was determined using a protocol described earlier [24]. A total of 500 μL of 0.052 M sodium pyrophosphate buffer (pH 8.3) was added to 500 μL of 186 μM PMS, 500 μL of 300 μM NBT and added to 50 μL sample, 500 μL dH_2_O and 500 μL of 780 μM NADH and incubated at 37 °C for 90 s. One mL of glacial acetic acid was added to stop the reaction. The mixtures were shaken vigorously with 4 mL of n-butanol and incubated for 10 min before being centrifuged. Butanol layers were separated and absorbance values were read at 560 nm.

#### Catalase (CAT) assay

The CAT assay was carried out following a described protocol [25], by adding 600 μL of 0.1 M PBS (pH 7.1) to 350 μL of 0.059 M hydrogen peroxide (H_2_O_2_) and 50 μL of sample. The absorbance was then read at 340 nm.

#### Glutathione reductase (GR) assay

The GR activity was determined according to a described method [26]. MilliQ water (855 μL) was added to 50 μL of 0.1 M PBS (pH 7.7), 50 μL of NADH, 20 μL of GSSG and 25 μL of sample. The absorbance was then read at 340 nm.

### Thin layer chromatography (TLC)

The most potent extract of each sample with the lowest IC_50_ (in the MTT assay) was chosen for partial purification through column chromatography. The best solvent system for column chromatography was determined by TLC. Solvent systems hexane : ethyl acetate; acetone : methanol and ethyl acetate : methanol were tested at ratios of 5: 5; 6: 4; 7: 3; 7.5: 2.5; 8: 2 and 9: 1.

### Partial purification of extracts by column chromatography

A glass column (32 cm length and 1.3 cm diameter) was packed with silica and hexane by the dry packing method. The column was equilibrated with hexane overnight to prevent cracking. 10 μL of sample was loaded and eluted with pure hexane. Subsequent elutions were carried out using solvents of higher polarity (hexane: ethyl acetate at 7.5: 2.5 and 5:5; pure ethyl acetate; acetone methanol at 9: 1; 8: 2; 7: 3 and 6: 4; and finally with pure methanol).

TLC was subsequently carried out with hexane: EA (7.5: 2.5). Fractions with the same retention factor (R_f_) were pooled for further analysis. MCF-7 cells were treated with the partially purified pooled fractions and subjected to the MTT assay to determine their effect on cell proliferation. Fractions with the lowest IC_50_ values were used for further analysis.

### Liquid chromatography-mass spectrometry (LC-MS) profiling

The fractions were subjected to LC-MS after testing for cytotoxic activity. Samples (500 μL) were injected and analysed with Agilent 1290 Infinity LC system coupled to Agilent 6520 Accurate-Mass Q-TOF mass spectrometer with dual ESI source. The dimension of the column used was 2.1 mm × 150 mm (3.5 μm). The binary mobile phase consisted of solvents A (0.1 % formic acid in water) and B (100 % acetonitrile with 0.1 % formic acid). The flow rate was 0.21 mL/min. Data were analysed with Agilent MasHunter Qualitative Analysis B.05.00 software. Compounds were identified by searching METLIN: Metabolite and Tandem MS Database.

The MS parameters were: nebulizer pressure, 45 psi; gas temperature, 300 °C; capillary voltage, 4000 V; fragmentor voltage, 125 V; gas flow, 10 μL/min. The electrospray ionization (ESI) source was set at positive and negative modes for acquiring all mass spectrometric data.

### Determination of caspase activities

MCF-7 cells were treated with the most potent fraction (those with the lowest IC_50_) and pure compounds for 8, 16, 24 and 45 h. The assay was carried out with the caspase-3, caspase-8 and caspase-9 Colorimetric Assay Kit (BioVision Inc., California, USA). Treated cells were counted and pelleted (1 × 10^6^ cells). Cell pellets were resuspended in 50 μL of chilled lysis buffer (from the kit: 150 mM sodium chloride, 1.0 % Triton X-100, 0.5 % sodium deoxycholate, 0.1 % SDS and 50 mM Tris, pH 8.0). The suspensions were incubated in ice for 10 min and subsequently centrifuged at 10,000 × g for 1 min. The supernatants were stored at −80 °C for further analysis. Protein concentrations were quantified using the Bradford assay. A total of 50 μg of protein (as estimated by the Bradford assay) was diluted with 50 μL of cell lysis buffer. Dithiothreitol (DTT) (10 μL) was added to 1 mL of 2X reaction buffer (from the kit). 2X reaction buffer (50 μL) and 5 μL of substrate were added and incubated for 2 h. Samples were diluted with dilution buffer and the absorbance taken at 405 nm.

### DNA fragmentation assay

MCF-7 cells were treated with the most potent fraction of each sample for 24 and 48 h. The DNA of the cultured cells was then extracted with Tri Reagent. Samples were electrophoresed at 80 V for 2 h by 0.6 % agarose gel electrophoresis.

### Statistical analysis

For all experiments, data were reported as mean ± standard error (SEM) (*n* = 3) of data obtained from triplicate experiments using SPSS software. Statistical analyses were performed by one-way analysis of variance (ANOVA) with Tukey’s multiple comparisons and the Student’s *t*-test. The correlation between TPC and DPPH radical scavenging activity of each extract was assessed by the Pearson correlation tests and the level of significance was tested by Student’s *t*-test (*p* < 0.05). A *p*-value of less than 0.05 or 0.01 was considered significant. SPSS, version 18.0 (Chicago, Ill, USA) and Microsoft Excel 2010 (Roselle, Ill, USA) statistical software were used for the statistical and graphical evaluations.

## Results and discussion

### Cytotoxic activity

The seaweeds were extracted in five different solvents, i.e., hexane, dichloromethane (DCM), ethyl acetate (EA), acetone and methanol. The cytotoxic activity of each extract on MCF-7 cells was determined. As shown in Table 1, the methanolic extracts of *C. racemosa* and *T. ornata* had the lowest IC_50_ values. For *P. tetrastromatica*, only the hexane extract showed an IC_50_ value. Among all samples tested, *T. ornata* showed cytotoxic activity with most of the solvents, compared with *P. tetrastromatica* and *C. racemosa*. Thus, the hexane extract of *P. tetrastromatica* and methanolic extracts of *C. racemosa* and *T. ornata* were chosen for partial purification through column chromatography. No cytotoxicity was observed in extract-treated 184B5 cells. MCF-7 and 184B5 cells were treated with doxorubicin as a positive control. The drug showed IC_50_ values at 0.21 ± 1.25 μg/ml and 0.02 ± 1.97 μg/ml in MCF-7 and 184B5 cells, respectively.

**Table 1:** Cytotoxic activity of different solvent extracts of *P. tetrastromatica*, *C. racemosa* and *T. ornata* in human adenocarcinoma MCF-7 cells and the human non-tumourigenic 184B5 cells

Extracts	MCF-7 cells IC_50_ (μg/mL)			184B5 cells IC_20_ (μg/mL)		
	*P. tetrastromatica*	*C. racemosa*	*T. ornata*	*P. tetrastromatica*	*C. racemosa*	*T. ornata*
Hexane	130.0 ± 1.72	ND	240.0 ± 1.89	280.5 ± 1.34	ND	ND
Dichloromethane (DCM)	ND	ND	ND	ND	ND	ND
Ethyl acetate (EA)	ND	ND	ND	ND	ND	ND
Acetone	ND	100.0 ± 2.41	480.5 ± 2.27	ND	240.0 ± 1.26	ND
Methanol	ND	60.0 ± 1.47	60.0 ± 1.14	ND	150.5 ± 1.48	950.0 ± 0.62

Cytotoxic activity is expressed as IC_50_ (μg/mL) ± SD (*n* = 3) and IC_50_ (μg/mL) ± SD (*n* = 3) for MCF-7 and 184B5 cells, respectively, which is the concentration of extract at which 50 % of cell growth was inhibited relative to cells incubated in the presence of 0.1 % DMSO vehicle control. MCF-7 and 184B5 cells were treated with doxorubicin as a positive control. The drug showed IC_50_ values at 0.21 ± 1.25 μg/ml and 0.02 ± 1.97 μg/ml in MCF-7 and 184B5 cells, respectively

ND: Not detected

The hexane extract of *P. tetrastromatica* was the only extract that was able to achieve IC_50_ among all the five solvents tested. Hence, it was postulated that MCF-7 cells were more susceptible to the effects of the hexane extracts of *P. tetrastromatica* than the other solvents. However, in the case of *C. racemosa* and *T. ornate*, the methanolic extracts of both seaweeds exhibited greater potency among the five solvents tested. Thus, it is suggested that MCF-7 cells are more susceptible to the effects of polar compound(s) from the methanolic extracts of *C. racemosa* and *T. ornata* as methanol is a polar solvent. The susceptibility and resistance of MCF-7 cells to different extracts vary as the compound(s) present in all the marine organisms have variable polarity and extractability [27]. From the results obtained, the seaweed extracts were more suited to be used as chemotherapeutic agents as compared with the standard drug, doxorubicin. This is because treatment with doxorubicin in normal breast cells (184B5) resulted in high toxicity to the cells.

The cytotoxic activity of the extracts can be attributed to the presence of active phytochemicals such as quinine and alkaloids. Quinine derivatives confer cytotoxic activity via interference of DNA and RNA replication and mitochondrial oxidative pathways, as well as through the formation of peroxide, superoxide and hydroxyl radicals in the cell. Cytotoxic activity of alkaloids, on the other hand, is due to the presence of microtubule interfering agents that can bind to beta tubulin, thus inhibiting the formation of the mitotic spindle fibre required for cell division [28].

### Estimation of total phenolic content (TPC)

The methanolic extract of *P. terastromatica* and *C. racemosa*; and the DCM extract of *T. ornata* showed the highest percentage yield among all five solvents tested, with yields of 8.71 %, 0.75 % and 4.08 %, respectively. The lowest percentage yield of *P. tetrastromatica*, *C. racemosa* and *T. ornata* was in hexane (0.04 %), EA (0.04 %) and EA (0.05 %), respectively. However, the acetone extract of *T. ornata* had the highest TPC with a value of 71.3 ± 2.14 mg GAE/g dried weight, despite of its low percentage yield. The methanolic extracts of *P. tetrastromatica* and *C. racemosa* demonstrated the highest TPC among the five solvents used for each seaweed sample, with TPC values of 69.5 ± 1.74 and 19.8 ± 2.01 mg GAE/g dried weight, respectively (Table 2).

**Table 2:** IC_50_ values in free radical scavenging assays for extracts of *P. tetrastromatica*, *C. racemosa* and *T. ornata*

	*P. tetrastromatica*	*C. racemosa*	*T. ornata*	Quercetin	Rutin	Catechin
Total phenolic content (mg GAE/g)	69.5 ± 1.74 (Methanol extract)	19.8 ± 2.01 (Methanol extract)	71.3 ± 2.14 (Acetone extract)	-	-	-
Total flavonoid content (mg of catechin equivalents/g dried weight)	38.4 ± 1.64 (Methanol extract)	16.0 ± 0.52 (EA extract)	17.5 ± 1.06 (Hexane extract)	-	-	-
IC_50_ of DPPH radical scavenging activity (μg/mL)	171.67 ± 2.89 (EA extract)	90.00 ± 0 (Hexane extract)	280.67 ± 1.15 (Methanol extract)	21.67 ± 2.89	23.33 ± 2.89	-
IC_50_ of superoxide anion scavenging activity (μg/mL)	ND	ND	20.00 ± 0 (EA, acetone, methanol extracts)	260.00 ± 0	223.33 ± 2.89	-
IC_50_ of nitric oxide scavenging activity (μg/mL)	ND	38.33 ± 2.89 (DCM extract)	25.00 ± 0 (DCM and acetone extracts)	20.00 ± 0	20.00 ± 0	-
IC_50_ of hydroxyl radical scavenging activity (μg/mL)	23.33 ± 2.89 (Hexane extract)	20.00 ± 0 (Hexane and acetone extracts)	20.00 ± 0 (Methanol extract)	20.00 ± 0	101.67 ± 2.89	-

Quercetin and rutin were used as positive controls for all scavenging assays and for the determination of TPC whereas catechin was used as a positive control for the determination of TFC. Each value is expressed as mean ± SD (*n* = 3). Extracts in parentheses () indicate the solvent extracts with the highest TPC and TFC contents and best radical scavenging activities

*ND* Not detected

The variations in the TPC values obtained in this study might be due to the dilution of phenolic concentration per g extract by extracted matter other than phenolic compounds. This is because methanol is not only an effective solvent for the extraction of phenolics but also for the extraction of other compounds [29]. All three samples showed significantly higher TPC (ranging from 6.4 to 71.3 mg GAE/g) than the methanolic-chloroform, petroleum ether, ethyl acetate, butanol and aqueous extracts (ranging from 2.8 to 33.4 mg GAE/g) of five brown seaweeds [30].

Methanol is generally the most suitable solvent for the extraction of polyphenolic compounds due to its ability to inhibit the action of polyphenol oxidase that leads to the oxidation of polyphenols (Yermilo et al., [31]). Other studies have also reported methanolic extracts as having the highest TPC in edible Japanese brown seaweeds, three Nigerian plants and *Tecoma stans*, also known as yellow elder or small tree [29]. Methanolic extracts contain significantly high TPC as phenolic compounds are typically more polar compounds.

Generally, the two brown seaweeds, *P. tetrastromatica* and *T. ornate*, had higher TPC compared to the green seaweed, *C. racemosa*. This might be attributed to the presence of phlorotannins, bi-polar polyphenols commonly found in brown seaweeds. Phlorotannins show antioxidative properties due to the presence of multiple phenolic groups that assist the algae to overcome oxidative stress arising from their environment [29].

### Determination of total flavonoid content (TFC)

The TFC of the different solvent extracts of the three samples are shown in Table 2. The methanolic extract of *P. tetrastromatica* had the highest TFC (38.4 ± 1.64 mg/g dried weight) while the DCM extract of *C. racemosa* and *T. ornata* had the lowest TFC of 4.6 ± 0.06 mg/g dried mass, respectively. The acetone extract of *T. ornata*, on the contrary, demonstrated a relatively low TFC (8.1 ± 0.74 mg/g dried mass) despite having the highest TPC.

Flavonoids are the major subclass of polyphenols and antioxidants in plants. Generally, flavonoids occur as glycosides, containing several phenolic hydroxyl groups on their ring structures. Flavonoids are effective ROS scavengers due in part to the presence of phenolic hydroxyl groups [32]. It was postulated that flavonoids may have direct contribution to antioxidative actions. Antioxidant activity depends on the number and position of hydroxyl groups, other substituents and glycosylation of flavonoid molecules [33].

The acetone extract of *T. ornata* in this study showed relatively low TFC despite having a high TPC value, suggesting that flavonoids are not the most abundant polyphenols present in *T. ornata*, and the high TPC values might be due to the presence of other phytochemicals such as phenolic acids, stilbenes, lignans, alkaloids, essential oils, ascorbic acid, tocopherols, carotenoids and steroids [34].

The TFC values of the four marine organisms in the present study were higher than that of two seaweeds (*Ulva lactuca* and *Sargassum wightii*) with TFC values of 1.35 ± 0.04 and 2.02 ± 0.07 mg GAE/g, respectively [35]. As reported by Cox et al. [36], the TFC values for six species of edible Irish seaweeds were in the range of 6.83 – 52.50 mg/g, which is comparable to the TFC values of the samples in this study.

### Antioxidant assays

Phenolic compounds act as antioxidants via several pathways. The most significant pathway is the scavenging of free radicals in which free radical chain reactions can be interrupted by phenolic compounds. In addition, polyphenols demonstrate antioxidant activity by the inactivation of lipid radicals and by the prevention of degradation of hydroperoxides to free radicals. Antioxidant activities are predominantly attributed to their redox properties, allowing them to act as reducing agents, singlet oxygen quenchers and hydrogen donors.

#### 1,1-diphenyl-2- picrylhydrazyl (DPPH) radical scavenging activity

The hexane extract of *C. racemosa* showed the lowest IC_50_ value of 90.00 ± 0 μg/mL whilst the IC_50_ value for the methanol extract of *T. ornata* was the highest (280.67 ± 1.15 μg/mL) as summarized in Table 2.

The free radical scavenging assay using the DPPH radical is a preliminary test for the analysis of the antioxidant potential of extracts. The assay has been used extensively as it allows high throughput screening and it has high sensitivity for the detection of active ingredients even at low concentrations. Antioxidant activity is related to the presence of bioactive compounds such as phenolics, flavonols and flavonoids. Polyphenols and anthocyanins scavenge DPPH via the donation of hydrogen, thus reducing DPPH (DPPH-H). Since the DPPH radical scavenging activity of the seaweeds in our study is considerable, they could be used as substitutes to replace harmful synthetic antioxidants commonly used in processed food products, such as butylated hydrotoluene (BHT) and butylated hydroxyanisole (BHA), which have been reported to be carcinogenic and tumorigenic at high doses [37]. In our study, all three samples demonstrated higher DPPH radical scavenging activity (ranging from 10.96 ± 0.81 % to 75.17 ± 2.04 %) compared to the methanolic-chloroform, petroleum ether, butanol and aqueous extracts (ranging from 6.08 ± 0.32 % to 58.25 ± 1.36 %) of five brown seaweeds from China [30]. This might be attributed to the different extraction methods used. As reported by Luo et al. [30], the mixture was sonicated for 2 h whereas the mixture in our study was macerated for 24 h.

#### Superoxide anion scavenging activity

The acetone extract of *T. ornata*, with the highest TPC, demonstrated the highest percentage of superoxide anion inhibition of 75.31 ± 0.29 %. At a concentration of 100 μg/mL, the superoxide anion scavenging activity of the five extracts of *T. ornata* (ranging from 55.23 to 66.41 %) was higher than that of the positive controls, quercetin and rutin (10.88 % and 35.67 %, respectively) (Table 2). Only the ethyl acetate, acetone and methanolic extracts of *T. ornata* demonstrated strong superoxide anion scavenging activity. The IC_50_ values of these extracts were significantly lower than that of the positive controls, indicating their higher potency in scavenging superoxide anions. Therefore, the antioxidant mechanism of these extracts might be mainly due to their superoxide anion scavenging ability.

Superoxide anion is the reduced form of molecular oxygen produced from the mitochondrial electron transport system upon the acceptance of a single electron. Energy is generated from mitochondria using electron transport chain reactions. Any loose electrons from the electron transport chain reactions will react directly with molecular oxygen, forming superoxide anion, which is the precursor for the formation of other ROS, including hydrogen peroxide, hydroxyl radicals and singlet oxygen [38]. *T. ornata* demonstrated stronger superoxide anion scavenging activity compared to the positive controls. This might be attributed to the presence of sulfated polysaccharides from *T. ornata* which have been reported to be excellent antioxidants for the management of oxidative stress [39].

#### Nitric oxide scavenging activity

The methanolic extract of *T. ornata* exhibited the highest percentage of inhibition of 54.96 ± 0.41 % but did not show any IC_50_. The DCM and acetone extracts of *T. ornata*, which showed lower IC_50_ values, on the other hand, exhibited a percentage of inhibition of around 53 %.

Nitric oxide (NO) is an important cellular messenger involved in numerous physiological functions of the body. Nitric oxide is generated from L-arginine by vascular endothelial cells, certain brain cells and phagocytes. The damage brought about by nitric oxide and oxygen free radicals is further exacerbated as they react to produce peroxynitrites, which in turn lead to severe toxic reactions in nucleic acids, proteins and lipids [31]. The NO generated from sodium nitroprusside (SNP) reacts directly with oxygen to produce nitrite. The results shown in Table 2 indicate that the dichloromethane extract of *C. racemosa* and the DCM and acetone extracts of *T. ornata* in SNP solution are capable of decreasing the levels of nitrite. The reduction in the nitrite level might be due to the direct competition between the extracts and oxygen in the reaction with NO. The DCM extract of *C. racemosa* and the DCM and acetone extracts of *T. ornata* had lower IC_50_ than the IC_50_ of ethyl acetate extract of *Cassia auriculata* leaves (51.3 μg/mL) [40].

##### Hydroxyl radical scavenging activity

Hexane and acetone extracts of *C. racemosa* and the methanol extract of *T. ornata* showed similar IC_50_ values (20.0 ± 0 μg/mL) to that of quercetin. The results in Table 2 show that all three samples exhibited strong hydroxyl radical scavenging activity, comparable to that of quercetin.

The hydroxyl radical is the most reactive radical and has the shortest half-life among other ROS due to its ability to induce severe damage to adjacent molecules. It can cause cell damage by reacting with lipids, saccaharides, polypeptides and nucleotides. In this study, EDTA, which acts as an iron chelator, was not used. Hence, hydroxyl radicals are generated site-specifically whereby unchelated iron ions are weakly associated with deoxyribose. These iron ions then react with hydrogen peroxide through the Fenton reaction, forming hydroxyl radicals that launch an immediate attack on the deoxyribose. When extracts are added to the reaction mixture, they remove the hydroxyl radicals from deoxyribose, thus directing the damage towards them and preventing the reaction [22]. The IC_50_ values indicate that these extracts are better hydroxyl radical scavengers than rutin. The three samples under study showed a higher percentage inhibition of hydroxyl radicals (ranging from 37.22 to 73.26 %) than the methanolic-chloroform, petroleum ether, EA, butanol and aqueous extracts of five brown seaweeds (ranging from 13.65 to 67.65 %) [30].

### Correlation between TPC and DPPH radical scavenging activity

The TPC of *P. tetrastromatica*, *C. racemosa* and *T. ornata* were found to be positively correlated with the DPPH radical scavenging activity (with Pearson correlation coefficient r values of 0.96, 0.73 and 0.93, respectively). Brown seaweeds (*P. tetrastromatica* and *T. ornata*) had higher TPC values compared with that of *C. racemosa.*

The Pearson correlation coefficients for *P. tetrastromatica* and *T. ornata* were similar, indicating that their DPPH radical scavenging activity might be attributed to their similar antioxidant content [41]. The DPPH scavenging activity of these brown seaweeds might be due to the presence of phlorotannins, the major antioxidants in brown seaweeds [29]. The scavenging activity of the green seaweed, *C. racemosa*, on the other hand, might be due to the presence of phytochemicals such as ascorbic acid, folic acid, Vitamin A and B1 [18].

### Antioxidant enzyme assays

**Fig. 1:**
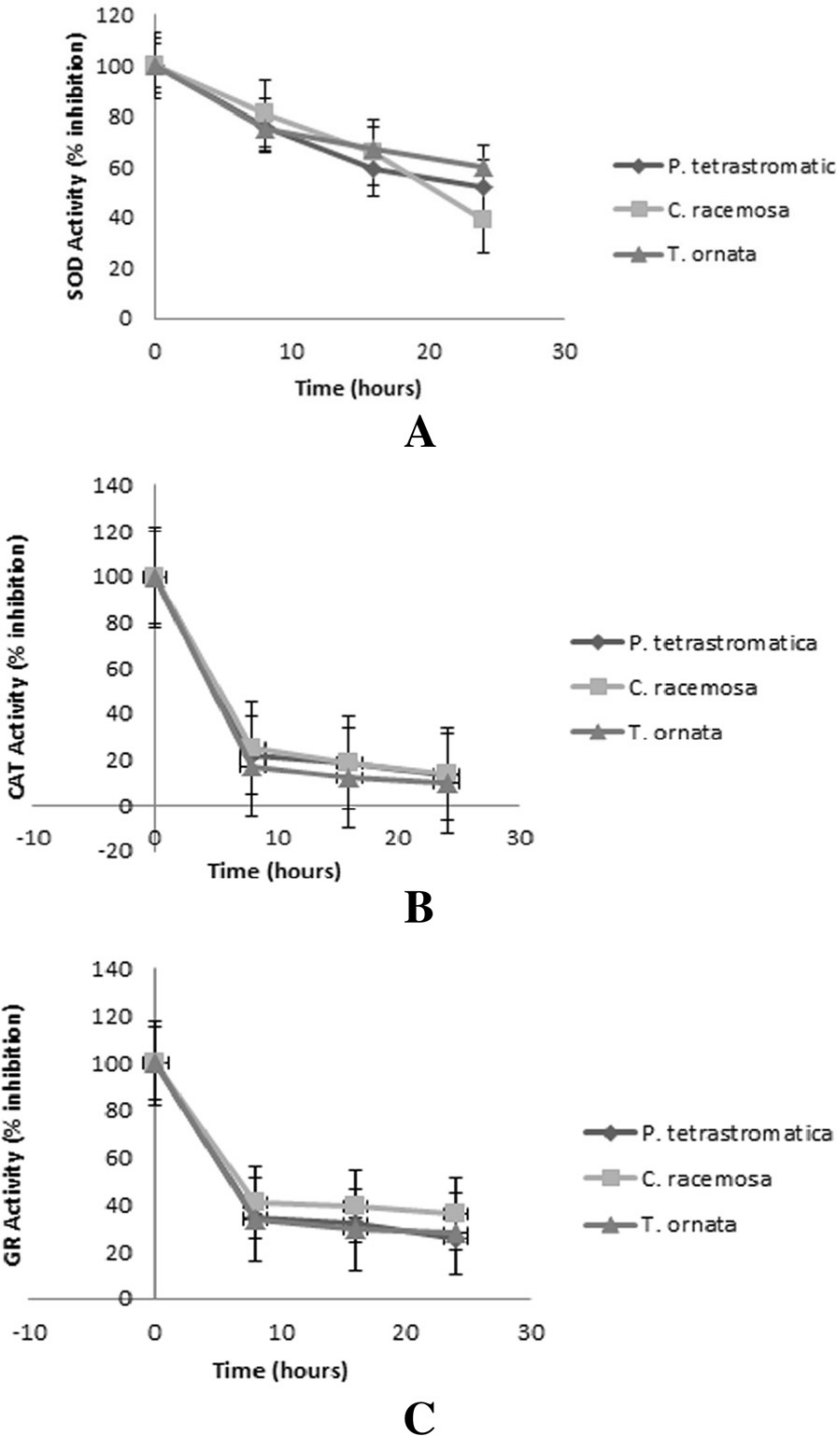
Antioxidant enzyme activities of methanolic extracts of *P. tetrastromatica*, *C. racemosa* and *T. ornate* for 8, 16 and 24 h. Untreated MCF-7 cells were used as the control and set as 100 %. Each value is expressed as mean ± SD (*n* = 3). **a** superoxide dismutase (SOD), **b** catalase (CAT), **c** glutathione reductase (GR). Note the enzyme activity decreasing upon treatment

Treatment of MCF-7 cells with 400 μg/mL of extract for 8, 16 and 24 h evoked a significant variation in the SOD, CAT and GR activities as compared to non-treated, control cells (Fig. 1). All three enzymes exhibited a decrease in activity relative to the control in a time-dependent manner. There was a sharp decrease in CAT activity within the first 8 h.

Antioxidant enzymes are involved in the direct elimination of ROS in cells. SOD constitutes the first line of defence against ROS in living cells. SOD catalyzes the dismutation of superoxide radical, which is the precursor of hydroxyl radicals and other ROS, to hydrogen peroxide (H_2_O_2_) and water. CAT, on the other hand catalyses the conversion of H_2_O_2_ to oxygen and water. GR facilitates the removal of H_2_O_2_ and organic peroxides [42]. Cancer cells generate excessive amounts of ROS, contributing to an altered redox status. These ROS include H_2_O_2_ and NO. An augmented level of ROS will lead to oxidative stress. One of the hallmarks of cancer cells as compared to normal cells is a persistent pro-oxidative state that can lead to intrinsic oxidative stress [43].

If the ROS produced could be removed immediately by free radical metabolizing enzymes (SOD, CAT and GR), normal cells might be protected against the cancer and carcinogenesis. The activities of SOD, CAT and GR for all three samples were found to be reduced in comparison to the untreated cells. The reduction in CAT activity is considered as a general response to stress. It has been suggested that the reduction in CAT activity is due to the inhibition of enzyme synthesis or alterations in the assembly of enzyme subunits under stress conditions [44]. A decrease in CAT and GR activities in treated MCF-7 cells might be due to increasing ROS. CAT can be downregulated by ROS while peroxides and hydroxyl radicals inactivate GR [45]. Accumulation of ROS due to the reduction in the enzyme activities might be the cause of cancer cell killing.

### Cytotoxic activities of partially purified fractions from bioassay guided fractionation

The partially purified fractions obtained from column chromatographic separation were subjected to the MTT assay. The cytotoxic activity of the partially purified fractions of all samples in MCF-7 cells increased significantly (Table 3). The column chromatographic separation of whole extracts yielded purer compounds which resulted in more potent cytotoxic activity.

**Table 3:** Cytotoxic activity of column chromatographic pooled fractions of the hexane extract of *P. tetrastromatica*, the methanolic extract of *C. racemosa* and the methanolic extract of *T. ornata* in the human adenocarcinoma MCF-7 cells and the human non-tumourigenic 184B5 cells

Sample	Pooled fractions	MCF-7 cells	184B5 cells
		IC_50_ (μg/mL)	IC_20_ (μg/mL)
*P. tetrastromatica*	P1	13.0 ± 1.68	120.0 ± 0.97
	P2	12.0 ± 2.01	138.5 ± 1.74
	P3	15.0 ± 1.73	125.0 ± 0.91
*C. racemosa*	P1	18.0 ± 1.43	105.0 ± 1.47
*T. ornata*	P1	12.0 ± 2.35	110.5 ± 1.95
	P2	22.0 ± 1.96	120.0 ± 1.47

Cytotoxic activity is expressed as IC_50_ (μg/mL) ± SD (*n* = 3) and IC_50_ (μg/mL) ± SD (*n* = 3) for MCF-7 and 184B5 cells, respectively, which is the concentration of extract at which 50 % of cell growth was inhibited relative to cells incubated in the presence of 0.1 % DMSO vehicle control. MCF-7 and 184B5 cells were treated with doxorubicin as a positive control. The drug showed IC_50_ values at 0.21 ± 1.25 μg/ml and 0.02 ± 1.97 μg/ml in MCF-7 and 184B5 cells, respectively

As shown in Table 3, there was significant enhancement of cytotoxic activity in the partially purified fractions of *P. tetrastromatica*, *C. racemosa* and *T. ornata* as compared to the crude extracts. A plausible explanation of this is because most unwanted components which are not bioactive, such as coloured pigments, resin or wax, were removed during sequential extraction, leaving only cytotoxic compounds or other bioactive phenolic compounds [46]. In addition, the existence of antagonistic interactions in the whole extracts could account for their weaker activities than those observed in the fractions [47]. Moreover, whole extracts may be inactive due to the fact that the active fractions or components may be present in minute quantities. Hence, their effects could have been suppressed by other compounds. However, the aforementioned compounds became concentrated and therefore exhibited higher activities upon fractionation [48].

### Liquid chromatography-mass spectrometry (LC-MS) profiling

**Fig. 2:**
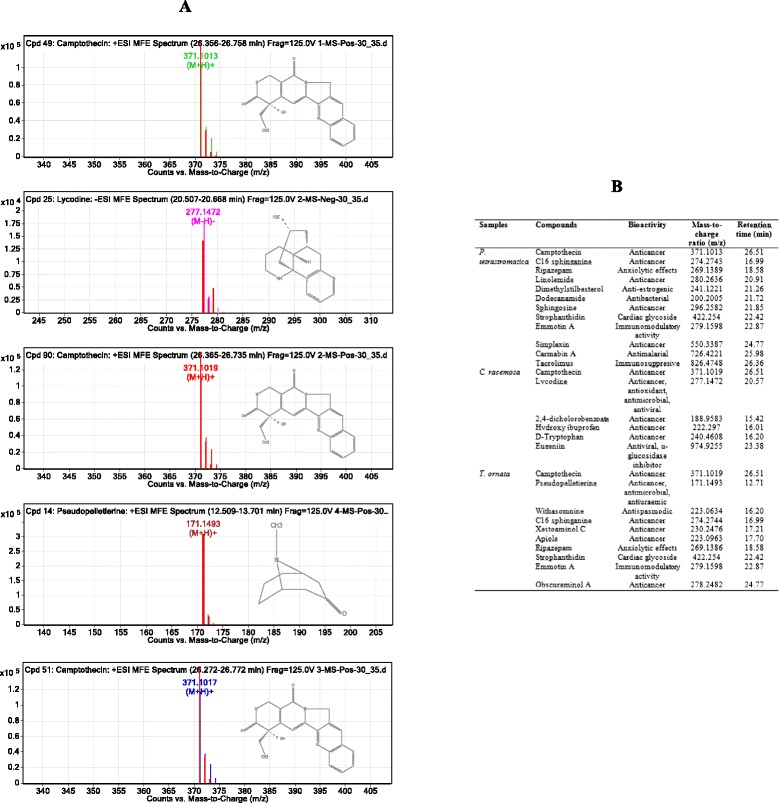
MS spectra of protonated and deprotonated molecules. **a** Camptothecin at [M + H] + of m/z 371; lycodine at [M-H]- of m/z 277; camptothecin at [M + H] + of m/z 371; pseudopelletierine at [M + H] + of m/z 171; Camptothecin at [M + H] + of m/z 371; Pseudopelletierine at [M + H] + of m/z 171; Camptothecin at [M + H] + of m/z 371. **b** Compounds with pharmacological activity isolated and identified in *P. tetrastromatica*, *C. racemosa* and *T. ornata* fractions

Following determination of cytotoxic activity, the pooled fractions P2 of *P. tetrastromatica*, P1 of *C. racemosa* and P1 of *T. ornata* were subjected to LC-MS analysis. Analysis of the mass spectral data for peaks at m/z 371 revealed the presence of camptothecin eluted at 26.5 min. Pseudopelletierine eluted at 12.7 min and lycodine at 20.5 min, showed fragments at m/z 171 and 277, respectively (Fig. 2a). The compounds detected are summarised in Fig. 2b.

Marine organisms such as fungi, bacteria, seaweeds and sponges are taxonomically diverse and biologically active, offering a wide array for the discovery of new anticancer drugs derived from bioactive compounds with medicinal properties such as terpenoid derivatives, flavonoids, flavones, alkaloids, glycosides, polyphenolics and steroids [49]. Alkaloids are widely present in natural products particularly in ethnobotanically important plants and marine organisms. Furthermore, alkaloids have been of specific interest due to their physiological activities including anticancer, antiviral and antimalarial activities [50]. LC-MS profiling revealed the presence of three important cytotoxic alkaloids in the seaweeds, which include camptothecin, lycodine and pseudopelletierine. Camptothecin (quinoline alkaloid) is one of the most well-known examples of cytotoxic alkaloids and accounts for approximately one-third of the global anticancer drug market [51]. Orhan et al. [52], reported that lycodine (tetracyclic alkaloid) isolated from the clubmoss, *Lycopodium clavatum* L., contributed to its antioxidant, antimicrobial and antiviral activities. Pseudopelletierine isolated from the stem bark and root of pomegranate was believed to have antimicrobial, anthelmintic and molluscicidal as well as antiuraemic activities [53]. Camptothecin, lycodine and pseudopelletierine had been reported to exhibit remarkable antioxidant activities such as DPPH, superoxide and hydroxyl radical scavenging activities [54–56]. Therefore, it is postulated that these compounds are the main contributors to the cytotoxic activity of the seaweeds in our study.

### Determination of caspase activities

**Fig. 3:**
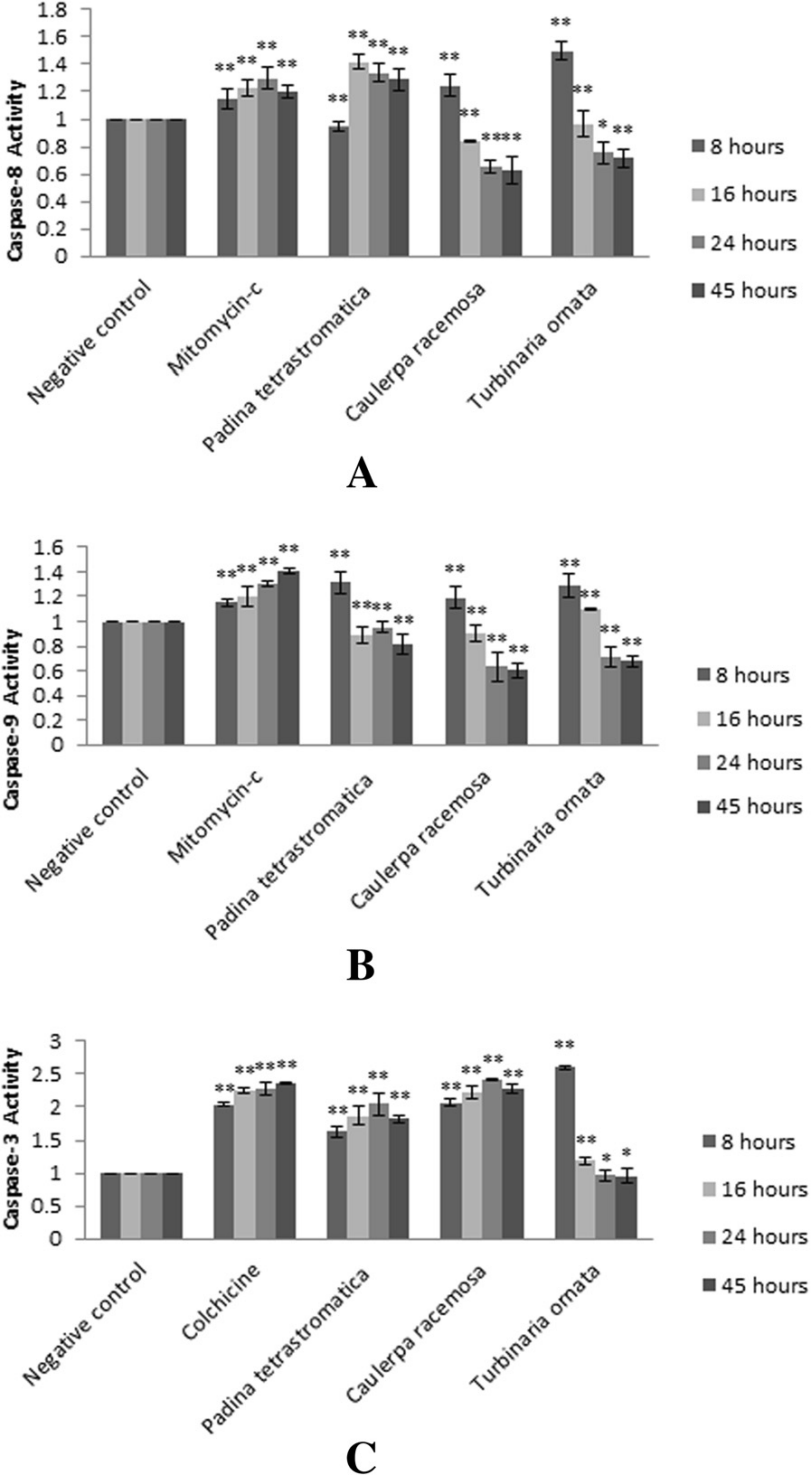
Caspase activities of MCF-7 cells treated with seaweed fractions. **a** Caspase-8, **b** Caspase-9 and **c** Caspase-3 activities of MCF-7 cells treated with 12 μg/mL of partially purified *P. tetrastromatica* column chromatographic fraction, 18 μg/mL of partially purified *C. racemosa* column chromatographic fraction and 12 μg/mL of partially purified *T. ornata* column chromatographic fraction. Mitomycin-c was used as a positive control for caspase-8 and 9 activities while positive control for caspase-3 was colchicine (*p < 0.05; **p < 0.01). Note caspase activity induction upon cell treatment

The caspase-8 activity of *P. tetrastromatica* increased and peaked at 16 h treatment by 1.4 fold, and then decreased while caspase-8 activities of *C. racemosa* and *T. ornata* peaked at 8 h treatment (by 1.2 and 1.5 folds, respectively) and then decreased (Fig. 3a). Caspase-9 activities peaked at 8 h treatment (by 1.3, 1.2 and 1.3 folds, respectively) and then decreased in a time-dependent manner for *P. tetrastromatica*, *C. racemosa* and *T. ornata* (Fig. 3b). Caspase-3 activities of *P. tetrastromatica* and *C. racemosa* increased and peaked at 24 h treatment (by 2.0 and 2.4 folds, respectively) and then decreased at 45 h treatment. Caspase-3 activities of *T. ornata*, on the other hand, peaked at 8 h treatment by 2.6 folds and then decreased in a time-dependent manner (Fig. 3c). In conclusion, the activities of caspases-8, −9 and −3 in treated cells increased over basal levels, indicating activation of these caspases in MCF-7 cells treated with the seaweed fractions.

It is hypothesized that the MCF-7 cells in this study died due to the cytotoxic effects exerted by the seaweed extracts. MCF-7 cell death might be induced by two mechanisms, necrosis and apoptosis. Necrosis is occasionally related to external damage, resulting in accidental cell death. Consequently, mitochondrial and cytoplasmic swelling, followed by compromised membrane integrity which subsequently leads to cell burst and release of cytoplasmic contents will occur [57, 58]. Apoptosis, on the other hand, involves a sequence of events, starting with the release of mitochondrial cytochrome c, activation of caspase cascades, degradation of poly-(ADP)-ribose polymerase (PARP) and eventually fragmentation of chromosomal DNA [59–61]. Caspase-3 plays a crucial role as early apoptosis biochemical markers in mammalian cells. The activity of the caspases studied suggests that the extracts inhibited the proliferation of MCF-7 cells by inducing apoptosis via both extrinsic (ligand-receptor mediated pathway) and intrinsic (mitochondrial mediated) pathways, since caspase-8, 9 and 3 were all activated. The mechanism by which caspase-8 is activated is unclear. However, since autoactivation induced by oligomerization can lead to activation of caspase-8, it could be possible that the samples induced oligomerization of caspase-8. In the extrinsic pathway, interaction of ligands, such as TNF or Fas, with their death receptors, Fas-associated death domain (FADD), leads to activation of initiator caspase (caspase-8). Executioner caspase (caspase-3) appears as a zymogen in cells and has no activity until it is cleaved by an initiator caspase (caspase-8 or 9) after being stimulated by apoptotic stimuli. Activated caspase-8 cleaves and activates caspase-3, which subsequently cleaves various substrates, leading to apoptosis [62]. In the intrinsic pathway, caspase-8 cleaves BID (pro-apoptotic member of Bcl-2 family) and causes the release of cytochrome c from the mitochondria. Cytochrome c together with apoptotic protease activating factor 1 (APAF1), ATP and procaspase-9 form an apoptosome, which then activate caspase-9. Caspase-9 in turn activates caspase-3, resulting in apoptosis [63].

### DNA fragmentation assay

**Fig. 4:**
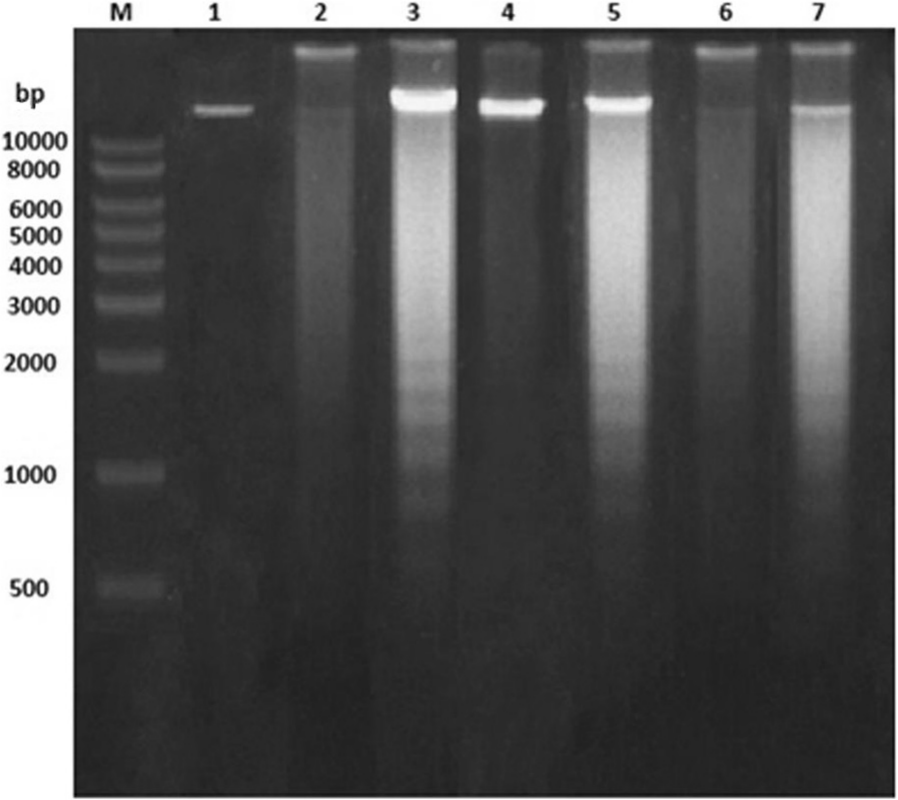
DNA fragmentation in MCF-7 cells treated with seaweed fractions. Lane M: 1000 bp DNA marker, Lane 1 : Untreated MCF-7 cells (negative control), Lanes 2 and 3: MCF-7 cells treated with 12 μg/mL of *P. tetrastromatica* for 24 and 48 h, respectively, Lanes 4 and 5: MCF-7 cells treated with 18 μg/mL of *C. racemosa* for 24 and 48 h, respectively, Lanes 6 and 7: MCF-7 cells treated with 12 μg/mL of *T. ornata* for 24 and 48 h, respectively

Figure 4 shows DNA degradation in extract-treated MCF-7 cells whereas the untreated control cells showed intact genomic DNA when observed with a UV transilluminator. Smearing pattern was observed in lanes 2, 4, and 6 (DNA from cells treated with *P. tetrastromatica*, *C. racemosa* and *T. ornata* for 24 h), whereas a typical laddering pattern was observed in lanes 3, 5 and 7 (treatment with the same samples for 48 h). This is because caspase-3 was activated at 24 h for *P. tetrastromatica* and *C. racemosa* and activated at 8 h for *T. ornata*.

DNA fragmentation is one of the hallmarks of apoptosis. The activity of caspase-3 is closely related to the initiation of DNA fragmentation. Following activation of caspase-3 upon treatment with the extracts, the proteolytic cleavage of caspase-3 substrates such as PARP and inhibitor of caspase activated DNase (ICAD) are triggered, leading to the release of the active endonuclease, caspase activated DNase (CAD) [59]. This endonuclease subsequently cleaves DNA during late apoptosis [64]. It is thus suggested that activation of caspase-3 is essential for DNA fragmentation during apoptosis. These DNA fragments were observed as DNA ladders after treatment with the extracts for 48 h on agarose gel as shown in Fig. 4, further validating that apoptosis was the cause of cell death of the MCF-7 cells.

## Conclusions

This study shows that the extracts of *P. tetrastromatica*, *C. racemosa* and *T. ornata* have high levels of antioxidants with free radical scavenging ability. These extracts showed higher superoxide and hydroxyl radical scavenging activities than the reference compounds (pure flavonoids, quercetin and rutin) despite having lower TPC and TFC values than the reference compounds. The partially purified fractions also demonstrated significant cytotoxic effects against MCF-7 cells. The antioxidant enzyme activities (SOD, CAT and GR) along with caspase activities and DNA fragmentation suggest that the extracts might induce cell death via apoptosis. The *in vitro* assays conducted in this study showed that the high cytotoxic and antioxidant activities of the three samples make it possible for the marine organisms to be used as sources of natural therapeutic antioxidants and dietary supplements for the prevention of and protection against damaging oxidative stress, chronic disease and maintenance of health and wellness.
